# Supplementary material for: Regulation of Tau Expression in Superior Cervical Ganglion (SCG) Neurons In Vivo and In Vitro
Source: Cells. 2023 Jan 5;12(2):226. doi: 10.3390/cells12020226 (PMC9856632; doi:10.3390/cells12020226)
Supplement: Supplementary file 1 [file cells-12-00226-s001.zip › cells-2061713-supplementary.pdf]

cells MDPI

Supplemental Images

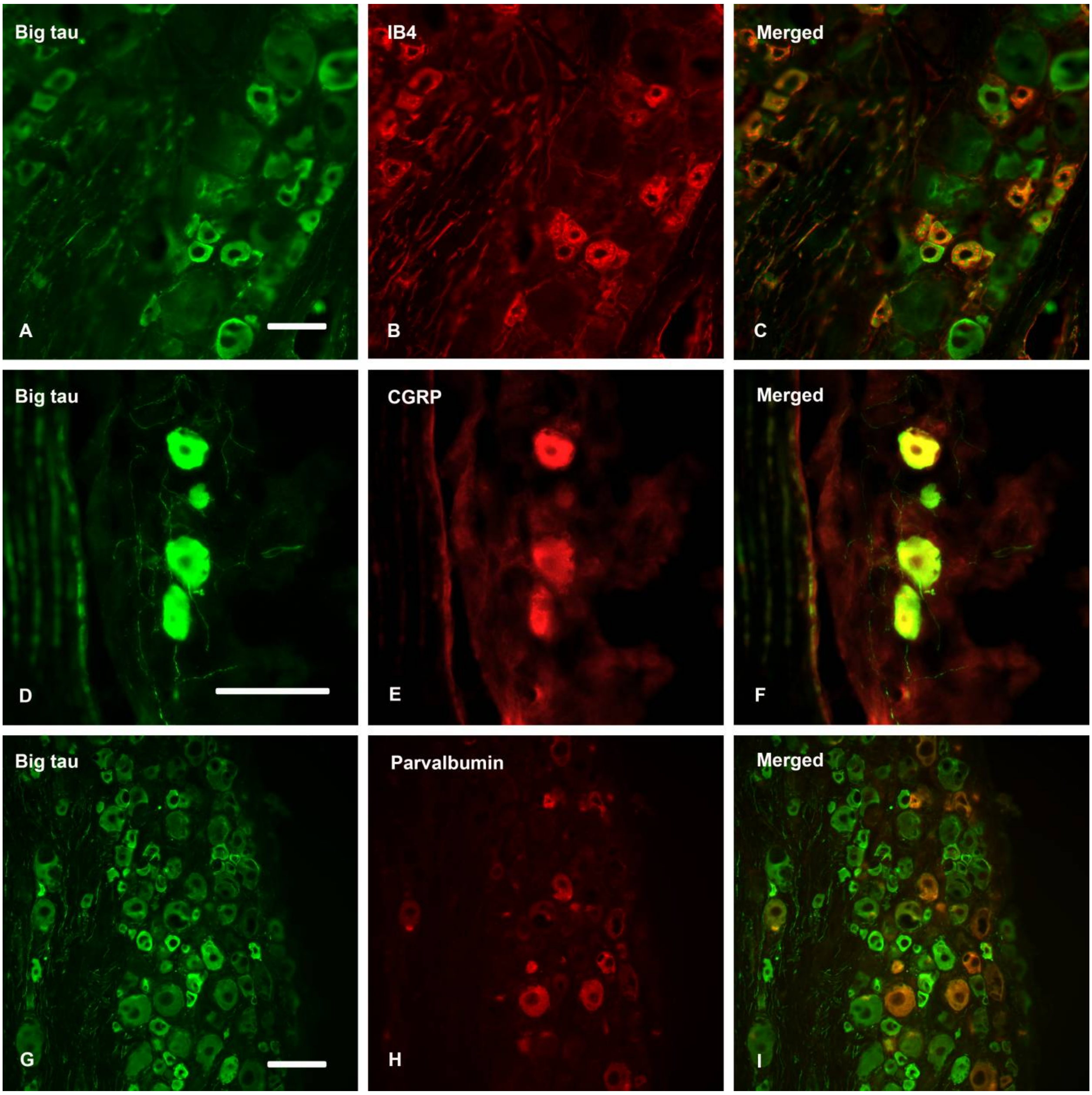

Figure. S1 Big tau (green) expression in the DRG co-stained with IB4, CGRP, and parvalbumin (red). Images A to C demonstrate co-localization of big tau with IB4 in a single and merged one. Images D to F show relationship of big tau and CGRP in a single and merged condition. Images G to I indicate the location of big tau and parvalbumin expression in the same DRG section. Scale bar = 100μm for all images.
